# Distraction in anterior plagiocephaly: technique, risks, and long-term results

**DOI:** 10.1007/s00381-026-07338-1

**Published:** 2026-08-04

**Authors:** Santiago Lopez-Becerra, David Perrault, Kirin Naidu, Maura R. Guyler, Samir Shakir, Jordan W. Swanson, Jesse A. Taylor

**Affiliations:** 1 Division of Plastic, Reconstructive and Oral Surgery, Children’s Hospital of Philadelphia, Philadelphia, PA 19104 USA; 2 Division of Plastic Surgery, Perelman School of Medicine at the University of Pennsylvania, Philadelphia, PA 19104 USA

**Keywords:** Unicoronal craniosynostosis, Fronto-orbital distraction osteogenesis, Fronto-orbital advancement and remodeling

## Abstract

**Purpose:**

The purpose of this study is to define the perioperative outcomes and long-term aesthetic outcomes of patients with unicoronal craniosynostosis (UCS) treated with fronto-orbital distraction osteogenesis (FODO) and compare them to a contemporaneous cohort treated with conventional fronto-orbital advancement and remodeling (FOAR).

**Methods:**

A retrospective review was performed for all patients treated at the Children’s Hospital of Philadelphia for non-syndromic UCS from 2009–2025. Demographic and perioperative data were collected. Preoperative and 5-year or greater postoperative frontal 2D photographs were used to assess for facial, and specifically periorbital, symmetry. Symmetry ratios were calculated to assess periorbital facial morphometry.

**Results:**

Sixty-two patients were included in the study, 34 FODO, and 28 FOAR. FODO demonstrated significantly shorter anesthesia and surgical times, lower estimated blood loss, and shorter length of stay (all *p* < 0.001). The overall complication rate was similar (*p* = 0.175), but postoperative infection was more commonly reported in the FODO group (24 vs 1%, *p* = 0.033). Thirty-two patients had a 5-year or greater postoperative photographs, 16 FODO, and 16 FOAR. Patients in the FODO group were found to have greater improvements in palpebral width symmetry (0.32 vs -3.4, *p* = 0.039) and canthal tilt symmetry (1.51 vs -1.20 degrees, *p* = 0.035). One patient in the FOAR group required reoperation due to signs of elevated ICP.

**Conclusion:**

FODO results in improved perioperative and long-term aesthetic outcomes compared to FOAR, resulting in improved canthal tilt and palpebral width symmetry. Future studies should focus on severity-matched analyses.

## Introduction

Craniosynostosis is the abnormal premature fusion of cranial sutures, which can result in impaired facial and skull growth and shape. Although underlying genetic mutations can cause syndromic forms of craniosynostosis, there are also multiple mechanical and environmental factors linked to non-syndromic craniosynostosis [1]. Unicoronal craniosynostosis (UCS) is determined by the premature fusion of one of the coronal sutures. It is also known as anterior plagiocephaly due to the frontal bone flattening observed on the affected side. Following sagittal and metopic, unicoronal craniosynostosis is the third most common type of craniosynostosis, with a prevalence rate ranging between 0.39 to 1.065 per 10,000 births, representing 6 to 17% of all cases of craniosynostosis [2–5]. UCS also has a higher prevalence in female patients with a 3:2 female-to-male ratio commonly reported in the literature [4, 6].

The diagnosis of UCS is typically made in the first year of life, by physical examination consisting of suture palpation and observation of facial morphometric changes. Diagnosis confirmation can be made through additional imaging, and although currently head CT is the most popular imaging modality, concerns for radiation exposure have led some groups to propose other methods such as black-bone MRI [7, 8]. Further, new machine learning algorithms such as CranioRate have been developed for automatic detection of craniosynostosis, although this is primarily for metopic and sagittal synostosis, and it has not been validated for unicoronal synostosis yet [9–11].

Amongst the different types of craniosynostosis, UCS presents one of the most challenging to treat due to its complex asymmetrical involvement of both face and skull [12]. Patients with UCS typically have aesthetic complications including orbital dystopia, frontal flattening, temporal retrusion, and nasal tip deviation on the affected side [13–16]. In severe cases, the affected orbital morphology may result in a harlequin deformity, which is characterized by a tall and narrow orbit on the affected side, and a wide orbit on the contralateral side [17, 18]. This alteration of normal orbital anatomy confers patients a higher risk of strabismus, astigmatism, and subsequent amblyogenic anisometropia, with rates of horizontal strabismus in UCS reported as high as 19% [19–21]. In addition, prior studies have reported that patients with untreated UCS are at risk of experiencing increased intracranial pressure (ICP) [22, 23].

In this context, surgical intervention for UCS aims to improve facial and skull morphology while also addressing functional ophthalmologic and neurologic outcomes. Fronto-orbital advancement and remodeling (FOAR) is the traditional treatment modality, and is typically performed between 6 and 12 months of age [24]. This procedure usually has a complication rate of 10–15%, although higher complication rates are seen with increased age [25]. Hemorrhage is the most common intraoperative complication of FOAR [25], and EBL can vary anywhere from 65 to 600 ml [26]. Further, rate of major complications is also usually low, but dural tears have been reported to occur between 0 and 33% of cases [27, 28]. FOAR also carries a long-term risk of fronto-orbital retrusion and temporal hollowing [16, 28], and a significant risk of strabismus [27, 29–32].

Minimally invasive options for UCS, such as endoscopic strip craniectomy, are becoming increasingly popular and have comparable short-term aesthetic outcomes [33]. Further, endoscopic suturectomy has shorter operative times, EBL, and length of stay compared with FOAR [27]. However, patients may later require revision surgeries and there is limited data on long-term outcomes [34]. Further, endoscopic suturectomy is usually reserved for younger patients under 5 months of age [27].

Fronto-orbital distraction osteogenesis has been popularized as an alternative solution in the past 15 years [35] resulting in improved long-term stability [36–40], improved ocular morphology [41], and lower rates of strabismus [42]. Further, minimally invasive modifications, such as endoscope-assisted FODO (endo-FODO) have lower morbidity and improved scarring [40, 43]. This study aims to provide our technique as well as an update on the long-term risks and outcomes of patients with UCS treated at our institution with FODO or FOAR.

## Patients and methods

### Study population

All patients with a diagnosis of non-syndromic UCS from 2009 to 2025 treated at the Children’s Hospital of Philadelphia were retrospectively reviewed. Patients were enrolled consecutively. A minimum of 30 days of follow-up was required to record all postoperative complications. Data collection included demographic, perioperative, and clinical data. This included total surgery and anesthesia time, estimated blood loss and transfusion volume, length of hospital stay, and short-term surgical complications. Preoperative and postoperative 2D facial photographs were retrieved from the patients records and analyzed to evaluate aesthetic outcomes. The ideal timing for each procedure performed is different. Therefore, patients undergoing FOAR typically undergo surgery at a later age.

### Surgical technique

#### FOAR

The approach for the FOAR that is employed at our institution has been previously reported [16]. It consists of a coronal exposure followed by bilateral frontal osteotomies 1 cm above the superior orbital rims and directly posterior the coronal sutures. This allows the creation of the bilateral frontal bone segment that will be mobilized. Then, an orbital bandeau is created. Earlier procedures were performed using a unilateral bandeau with unilateral reshaping. In later procedures, a classic two-thirds fronto-orbital bandeau was adopted. This is reconstructed along the superior orbital rim, reaching the contralateral orbit at the inflection point caused by the synostosis. Bone grafts are then harvested and placed at the temporal tenon or lateral orbit of the affected side, if needed, for additional stability of the orbital roof. In the contralateral superior orbital rim, an interpositional bone graft is placed to correct the horizontal dimensions of the affected orbit. Barrel stave osteotomies are then performed along the frontal bone. The final fronto-orbital bandeau and osteotomized forehead segment are fixated with resorbable plates and screws.

### FODO

#### Open FODO

**Fig. 1:**
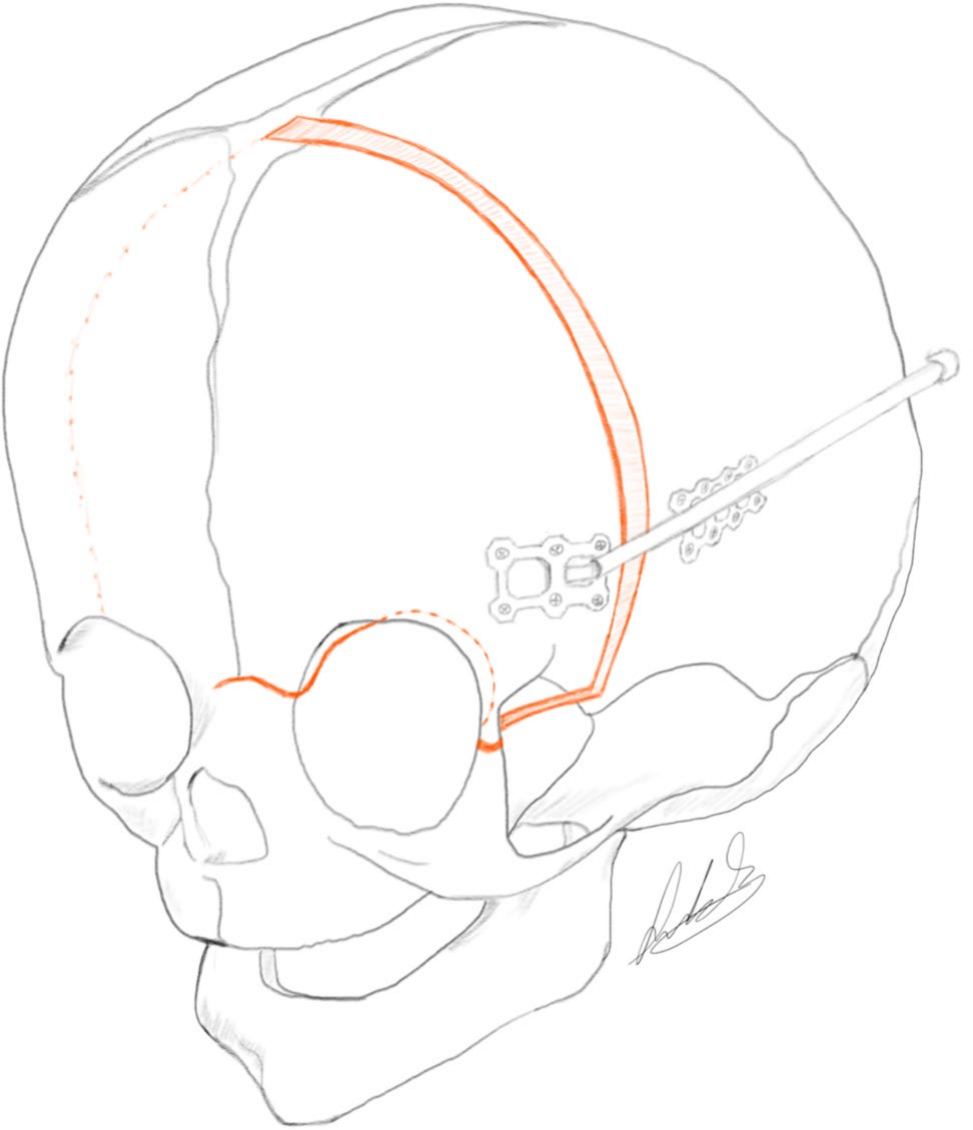
Illustration of our limited osteotomy pattern and distractor placement during open fronto-orbital distraction osteogenesis (Copyright © Santiago Lopez-Becerra)

The distraction method employed is based on a coronal incision followed by similar but limited osteotomy pattern compared to a traditional FOAR (Fig. 1**)**. No separate orbital bandeau is created during FODO, and a suturectomy is performed on the affected side [43, 44]. After a traditional coronal incision and appropriate subperiosteal dissection, a suturectomy of the affected suture is performed. A 1.5 to 2-cm-diameter pterional window is created at the base of the affected coronal suture, allowing release of the affected suture and access to the sphenoid wing and orbital roof. To preserve vascularity to the bone segments the dural dissection is kept to a minimum. Both the sphenoid wing and orbital roof are osteotomized under direct visualization, while retracting the brain with malleable retractors. Then a vertical osteotomy along the point of maximum frontal deformity is performed in the contralateral frontal bone. This serves as the hinge for the distraction of the ipsilateral frontal bone segment. Barrel stave osteotomies are performed when needed on the upper frontal bone region and over the ipsilateral orbit to allow for more flexible mobilization of the distracted segment. A linear distractor is then placed using resorbable pins with an anterior downward-pointing vector to allow improved projection of the ipsilateral forehead and downward mobilization of the ipsilateral orbital roof. Patients are then distracted at a rate of 1 mm/day starting on the second or third postoperative day and distracted until overcorrection is achieved.

#### Endo-FODO

**Fig. 2:**
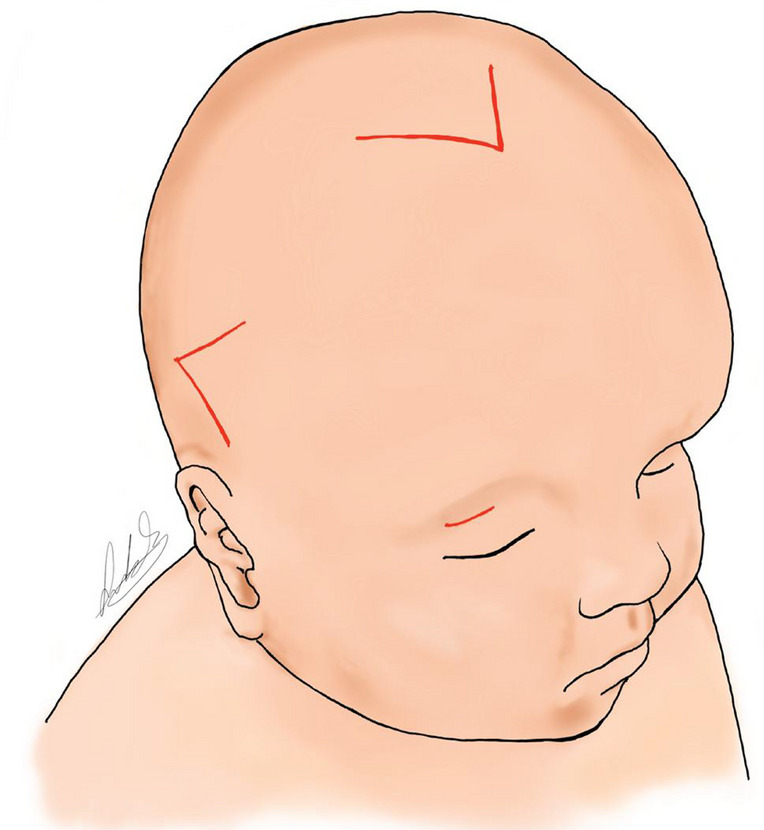
Illustration of the incision locations for Endo-FODO (Copyright © Santiago Lopez-Becerra)

The endoscopic (endo-FODO) variation of the procedure is performed through an ipsilateral upper lateral blepharoplasty incision, and 2 chevron incisions, one over the anterior fontanelle and another over the ipsilateral pterional region (Fig. 2). The temporalis muscle is released posteriorly through the pterional incision and remains attached anteriorly and along the temporal crest. A limited coronal suturectomy is then performed and a small 2-cm-diameter pterional window is created anterior to the suturectomy on the ipsilateral side. The endoscope is then employed for visualization of the sphenoid wing, and the ultrasonic scalpel is used to perform the osteotomies of the sphenoid wing and orbital roof with intracranial visualization. Periorbital dissection and visualization are performed via the lateral blepharoplasty incision. After all osteotomies are complete, a linear distractor is placed across the coronal suture using resorbable pins with anterior downward-pointing vector. Distraction is then performed following the same postoperative protocol as the open FODO procedure.

### Postoperative protocol

All patients underwent routine incision and scar care. No patients underwent postoperative helmeting or any other adjunctive measures. Patients undergoing FODO had weekly x-rays to monitor the progress of the distraction until culmination of the activation phase at 4 weeks postoperatively. Patients in the FOAR group did not undergo any additional considerations in the postoperative period.

### Photogrammetric analysis

Frontal facial photographs were collected from each patient at the preoperative (Pre) and latest postoperative follow-up (Post) period. Each photograph was annotated by 1 researcher using ImageJ (Rasband, W.S., ImageJ, U.S., National Institutes of Health, Bethesda, MD)[45] and following the same protocol previously described by Wu et al. [40]. The annotated landmarks included the nasion, columellar base, center of the pupil, medial canthus, lateral canthus, central point of the upper and lower eyelid borders, and central lower eyebrow border. These were used to calculate the margin-to-reflex distance 1 (MRD1), pupil-to-brow (PTB) distance, palpebral height (PH), palpebral width (PW), canthal tilt angle, and orbital dystopia angle (ODA), as previously defined by Villavisanis et al. [39]. The symmetry ratio formula [46] was utilized to assess overall symmetry between the affected (s) and unaffected (n) sides for each of the linear distance measurements:

$$symmetry ratio=|\left(1-\frac{s}{n}\right)x 100|$$

A symmetry ratio closer to zero indicated a more symmetric measurement, whereas a larger magnitude value indicated higher asymmetry. The change between preoperative and long-term symmetry ratios was defined such that a positive value indicated improved symmetry and a negative value indicated higher asymmetry.

### Statistical analysis

All data analysis was performed using R Statistical Software (v4.3.1; R Core Team 2023). Shapiro–Wilk test was used to assess for data normality. Chi-squared and Fisher’s exact tests were used to analyze categorical variables. Independent student’s T-tests or the Mann–Whitney U tests were performed to compare outcomes between different procedures.

## Results

A total of 62 patients were included in the study, 46 (74.2%) were female patients, 34 (54.8%) had right-sided UCS, and 48 (77.4%) identified as White. The FODO cohort had 34 (54.8%) patients, and the FOAR cohort had 28 (45.2%) patients. There were similar distributions for gender (*p* = 0.873), laterality (*p* = 0.557), and race (*p* = 0.178) between the FOAR and FODO cohorts. Patients in the FOAR group were older at surgery (median 8.8 months) compared with patients in the FODO group (median 5.5 months) (p < 0.001). The median follow-up time after surgery was 6.4 years (Min: 0.14, Max: 13.6) in the FOAR group and 4.9 years (Min: 0.28, Max: 10.3) in the FODO group (*p* = 0.302) (Table 1).

**Table 1:** Demographic characteristics of patients in the FOAR and FODO cohorts^a^

Variable	FOAR (*N* = 28)	FODO (*N* = 34)	P
Gender			0.873
Female	20 (71)	26 (76)	
Male	8 (29)	8 (24)	
Laterality			0.557
Left	11 (39)	17 (50)	
Right	17 (61)	17 (50)	
Race			0.178
African American	1 (4)	0 (0)	
Asian	4 (14)	1 (3)	
Other	1 (4)	5 (15)	
Unknown	1 (4)	1 (3)	
White	21 (75)	27 (79)	
Age at surgery (mo)	8.78 (7.89 to 10.87)	5.46 (4.98 to 6.26)	< 0.001
Weight at surgery (kg)	8.89 (7.96 to 9.66)	7.22 (6.75 to 7.64)	< 0.001
Follow-up time after surgery (yr)	6.4 (2.242 to 8.786) Min: 0.14, Max: 13.6	4.903 (1.896 to 7.76) Min: 0.28, Max: 10.3	0.302

^a^Values are given as median (IQR) or count (%)

*FOAR* fronto-orbital advancement and remodeling, *FODO* fronto-orbital distraction osteogenesis.

In the FOAR group, operative (median 187.5 min, IQR, 157.8 to 208.5 min) and anesthesia (median 294 min, IQR, 263.8 to 327.3 min) times were longer than in the FODO group (operative, median 110.5 min, *p* < 0.001; anesthesia, median 226 min, *p* < 0.001). Estimated blood loss (EBL) (median 13.9 ml/kg, IQR, 9.5 to 20.0 ml/kg) and blood transfusion requirements (median 20.8 ml/kg, IQR, 13.8 to 30.6 ml/kg) in the FODO group were significantly lower than in the FOAR group (EBL, median 24.6 ml/kg, *p* < 0.001; blood transfusion requirements, median 30.9 ml/kg, *p* = 0.020). Length of stay (LOS) was shorter in the FODO group at a median of 3 days than the FOAR group at median of 4 days (*p* < 0.001). The overall complication rate in the FODO group was 32% (11/34 patients) compared with 14% (4/28 patients) in the FOAR group (*p* = 0.1754). The FODO cohort also had a higher rate of postoperative infection at 24% (8/34 patients) compared with 4% (1/28 patients) in the FOAR cohort (*p* = 0.033); of note, all infections in both groups were treated with oral antibiotics with resolution. Three patients (11%) in the FOAR group had surgical site dehiscence, compared with no patients in the FODO group (*p* = 0.087) (Table 2).

**Table 2:** Perioperative complications associated with FOAR and FODO^a^

Variable	FOAR (*N* = 28)	FODO (*N* = 34)	P
Operative Time	187.5 (157.75 to 208.5)	110.5 (89.5 to 125)	< 0.001
Anesthesia Time	294 (263.75 to 327.25)	226 (207.75 to 266.75)	< 0.001
EBL/kg	24.550 (16.058 to 35.079)	13.944 (9.496 to 19.954)	< 0.001
Blood transfused/kg	30.919 (19.054 to 50.096)	20.817 (13.845 to 30.609)	0.020
Length of Stay (days)	4 (4 to 4)	3 (3 to 3)	< 0.001
30-day Readmission			0.620
No	27 (96)	31 (91)	
Yes	1 (4)	3 (9)	
30-day Reoperation			1.000
No	26 (93)	32 (94)	
Yes	2 (7)	2 (6)	
Infection			0.033
No	27 (96)	26 (76)	
Yes	1 (4)	8 (24)	
Dehiscence			0.087
No	25 (89)	34 (100)	
Yes	3 (11)	0 (0)	
Hardware Complications			0.497
No	28 (100)	32 (94)	
Yes	0 (0)	2 (6)	
CSF Leak			0.585
No	26 (93)	33 (97)	
Yes	2 (7)	1 (3)	
Any Complication			0.175
No	24 (86)	23 (68)	
Yes	4 (14)	11 (32)	
Subsequent Frontal Surgery			0.452
No	27 (96)	34 (100)	
Yes	1 (4)	0 (0)	

^a^Values are given as median (IQR) or count (%)

*FOAR* fronto-orbital advancement and remodeling, *FODO* fronto-orbital distraction osteogenesis.

### Subgroup long-term analysis

A total of 32 patients, 16 (50.0%) in the FOAR group and 16 (50.0%) in the FODO group, had a follow-up equal to or greater than 5 years. Median time from surgery to long-term clinical photograph was 7.13 years (IQR, 6.43 to 9.45 years) in the FOAR group, and 7.27 years (IQR, 5.55 to 8.62) in the FODO group (*p* = 0.289). These cohorts had a similar distribution of gender (*p* = 0.685), laterality (*p* = 0.479) and race (*p* = 0.223) (Table 3).

**Table 3:** Demographic characteristics of patients with 5-year or greater postoperative photographs^a^

Variable	FOAR (*N* = 16)	FODO (*N* = 16)	P
Gender			0.685
Female	11 (69)	13 (81)	
Male	5 (31)	3 (19)	
Laterality			0.479
Left	6 (38)	9 (56)	
Right	10 (62)	7 (44)	
Race			0.226
Asian	3 (19)	0 (0)	
White	13 (81)	15 (94)	
Other	0 (0)	1 (6)	
Age at surgery (mo)	8.61 (7.74 to 10.67)	5.458 (4.94 to 5.94)	< 0.001
Weight at surgery (kg)	8.77 (7.64 to 9.41)	7.047 (6.90 to 7.52)	0.003
Age at long-term photograph (yr)	8.07 (7.33 to 10.09)	7.723 (6.13 to 8.99)	0.114
Time from surgery to long-term photograph (yr)	7.13 (6.43 to 9.45) Min: 6.10, Max: 13.61	7.27 (5.55 to 8.62) Min: 5.19, Max: 10.30	0.289

^a^Values are given as median (IQR) or count (%)

*FOAR*, fronto-orbital advancement and remodeling. *FODO*, fronto-orbital distraction osteogenesis.

In photogrammetric analysis, patients in the FODO group demonstrated a greater improvement in their palpebral width symmetry at a median of 0.32 (IQR, −3.51 to 3.89), compared to the FOAR group with a median of −3.38 (IQR, −8.62 to 0.51) (*p* = 0.039). Patients in the distraction cohort also had a greater improvement in their canthal tilt symmetry (median 1.5 degrees, IQR −1.1 to 3.2 degrees versus median −1.2, IQR, −2.8 to 0.0, *p* = 0.035). No significant differences were observed between the FODO and FOAR cohorts in the MRD symmetry (*p* = 0.733), PTB symmetry (*p* = 0.880), palpebral height symmetry (*p* = 0.498), or ODA angle (*p* = 0.557). At the latest follow-up, one patient (6%) in the FOAR group required reoperation due to signs of elevated ICP, while no patients in the FODO group required reoperation for this reason. The patient that required reoperation was experiencing recurrent persistent headaches that resolved after undergoing a second FOAR nearly three years after the first procedure (Table 4).

**Table 4:** Photogrammetric outcomes of patients in FOAR and FODO cohorts before surgery and after 5-year follow-up^a^

Photogrammetric Variable	FOAR (*N* = 16)	FODO (*N* = 16)	P
Pre MRD Symmetry	−31.82 (−45.22 to −10.12)	−23.726 (−43.38 to −12.18)	0.720
Post MRD Symmetry	−8.12 (−18.29 to −2.32)	−8.083 (−14.29 to −4.03)	0.748
Change in MRD Symmetry	18.66 (1.02 to 41.33)	17.474 (5.06 to 31.43)	0.733
Pre PTB Symmetry	−18.9 (−27.30 to −9.8)	−17.582 (−25.53 to −11.40)	0.880
Post PTB Symmetry	−3.38 (−10.12 to −1.64)	−5.087 (−11.33 to −2.08)	0.546
Change in PTB Symmetry	12.19 (6.51 to 16.74)	10.813 (5.27 to 22.96)	0.880
Pre Palpebral Height Symmetry	−11.59 (−27.96 to −2.79)	−18.108 (−37.34 to −7.32)	0.428
Post Palpebral Height Symmetry	−3.70 (−11.16 to −2.51)	−4.373 (−9.30 to −0.97)	0.734
Change in Palpebral Height Symmetry	7.73 (−2.07 to 19.03)	16.049 (−0.83 to 27.89)	0.498
Pre Palpebral Width Symmetry	−4.87 (−6.88 to −2.47)	−5.921 (−8.15 to −3.18)	0.175
Post Palpebral Width Symmetry	−7.44 (−11.57 to −3.81)	−5.159 (−8.43 to −4.16)	0.274
Change in Palpebral Width Symmetry	−3.38 (−8.62 to 0.51)	0.316 (−3.51 to 3.89)	0.039
Pre Canthal Tilt Symmetry	2.45 (1.69 to 5.06)	3.732 (2.13 to 7.60)	0.291
Post Canthal Tilt Symmetry	3.83 (2.79 to 6.09)	2.761 (0.30 to 4.59)	0.122
Change in Canthal Tilt Symmetry	−1.12 (−2.80 to 0.02)	1.502 (−1.07 to 3.23)	0.035
Pre ODA	83.24 (82.22 to 84.69)	83.182 (81.54 to 85.14)	0.970
Post ODA	87.53 (85.71 to 88.95)	88.417 (87.23 to 88.67)	0.557
Change in ODA	5.2 (2.89 to 6.35)	3.595 (2.40 to 6.95)	0.910

^a^Values are given as median (IQR) or count (%)

*FOAR* fronto-orbital advancement and remodeling, *FODO* fronto-orbital distraction osteogenesis.

### Open FODO vs. endo-FODO

Patients in the endo-FODO group were found to have a lower estimated blood loss (EBL) (9.43 ml/kg) when compared to the open FODO group (19.61 ml/kg) (*P* = 0.001). Endo-FODO has only been employed more recently, consequently, the endo-FODO group does not yet have as long-term follow-up as the open FODO group (1.4 years vs. 7.26 years for latest postoperative photograph, *p* < 0.001). One patient in the endo-FODO group has greater than 5 years of follow-up. Outcomes between endo-FODO and open FODO are included for completeness, however, we do not yet have sufficient data to compare long-term photogrammetric outcomes (Table 5).

**Table 5:** Perioperative complications associated with open FODO and endo-FODO cohorts^a^

Variable	Endo-FODO	Open FODO	P
Gender			0.688
Female	12 (71)	14 (82)	
Male	5 (29)	3 (18)	
Laterality			0.493
Left	7 (41)	10 (59)	
Right	10 (59)	7 (41)	
Race			1.000
Asian	1 (6)	0 (0)	
Other	2 (12)	3 (18)	
Unkown	1 (6)	0 (0)	
White	13 (76)	14 (82)	
Age at Surgery (mo)	5.458 (4.964 to 6.312)	5.458 (5.03 to 6.115)	0.756
Weight (kg)	7.19 (6.525 to 8.185)	7.25 (6.98 to 7.57)	0.828
Operative Time	104 (87 to 117)	117 (92 to 126)	0.230
Anesthesia Time	210 (190 to 237)	246 (218 to 270)	0.057
EBL/kg	9.434 (7.463 to 13.333)	19.608 (14.094 to 21.739)	0.001
Blood transfused/kg	15.094 (0 to 20)	31.008 (24.679 to 38.999)	< 0.001
Length of Stay (days)	3 (2 to 3)	3 (3 to 3)	0.373
30-day Readmission			1.000
No	16 (94)	15 (88)	
Yes	1 (6)	2 (12)	
30-day Reoperation			0.485
No	17 (100)	15 (88)	
Yes	0 (0)	2 (12)	
Infection			0.688
No	14 (82)	12 (71)	
Yes	3 (18)	5 (29)	
Dehiscence			1.000
No	17 (100)	17 (100)	
Yes	0 (0)	0 (0)	
Hardware Complications			0.485
No	17 (100)	15 (88)	
Yes	0 (0)	2 (12)	
CSF Leak			1.000
No	16 (94)	17 (100)	
Yes	1 (6)	0 (0)	
Any Complication			0.464
No	13 (76)	10 (59)	
Yes	4 (24)	7 (41)	
Subsequent Frontal Surgery			1.000
No	17 (100)	17 (100)	
Yes	0 (0)	0 (0)	
Follow-up time after surgery (yr)	1.88 (1.21 to 2.86) Min: 0.28, Max: 9.14	7.61 (5.56 to 8.99) Min: 3.27, Max: 10.30	< 0.001
Age at long-term photograph (yr)	2.06 (1.33 to 2.83)	7.62 (6.02 to 8.95)	< 0.001
Time from surgery to last photograph (yr)	1.41 (0.67 to 2.46) Min: 0.26, Max: 5.15	7.26 (5.55 to 8.61) Min: 0.08, Max: 10.30	< 0.001

^a^Values are given as median (IQR) or count (%)

*FODO* fronto-orbital distraction osteogenesis, *Endo-FODO* endoscope-assisted FODO.

## Discussion

Unicoronal craniosynostosis presents unique treatment challenges related to its asymmetrical skull involvement and facial scoliosis, and there is currently no consensus for the best treatment modality. FODO has previously shown better perioperative and long-term results in various facial anthropometric assessments when compared with FOAR [39, 40]. In our analysis of long-term outcomes, we demonstrate that FODO consistently results in improved operative times, estimated blood loss, LOS, and long-term outcomes. Further, endo-FODO results in lower estimated blood loss when compared to open FODO. Regarding long-term outcomes, we found greater improvements in canthal tilt and palpebral width symmetry with distraction osteogenesis compared to fronto-orbital advancement.

### Surgical complications and surgical techniques

In a systematic literature review Corkum and colleagues [47] found that patients undergoing FODO had better Whitaker aesthetic outcomes, but no significant difference was found in reoperation or infection rates. The results from our group also support better aesthetic outcomes for the FODO group [40]. However, we found a greater rate of infection in the distraction group (24%) compared to the FOAR group (*p* = 0.033). This underscores that distraction osteogenesis has many benefits regarding shorter procedures and LOS, but it is important to be aware of the risk of having an externalized device, especially as it relates to infection and/or device malfunction.

Other groups [36, 37] using a similar FODO technique we report also found better perioperative outcomes of FODO vs FOAR. They found that mean operative time was shorter for FODO at 89.5 min vs 153.5 min for FOAR. Their EBL for FODO (6.1 ml/kg) was lower than ours (13.9 ml/kg), but their LOS was longer (4.3 days vs. 3.0 days in our cohort). In their study, they also reported successful distraction even with older patients 13 to 17 months old. This is also in alignment with the previous results shown by our group [42, 46].

Other teams have also adopted FODO as an alternative for addressing UCS. Park and colleagues [38, 41], presented their experience with one-piece FODO, which consisted of a bilateral coronal osteotomy pattern, similar to traditional FOAR. In their early reports they found no significant differences in orbital height or width comparing the pre and postoperative periods. They noted a 2 mm preoperative difference in orbital width between the affected and unaffected sides in patients with UCS. They noted that this difference remained largely unchanged after their one-piece FODO. Leading them to conclude there was no improvement on orbital width symmetry. We believe this might have been due to their specific distraction pattern that resulted in a symmetric advancement and may have not corrected orbital dysmorphology. In their latest update [48], they reported implementing a hemi-one-piece FODO, which is similar to our technique, and found better outcomes than with a regular one-piece FODO. This also included a lower intraoperative transfusion product use, and shorter procedure duration.

A new promising option presented by Frommer and colleagues [49] is spring-assisted cranioplasty. Their hemicoronal incision approach results in an EBL of 21.7 cc/kg, comparable to our FOAR group (24.6 cc/kg), but higher than our FODO group (13.9 cc/kg). Their spring placement (83.1 min) and removal (75.4 min) times are quicker than our FODO (110.5 min) and FOAR (187.5 min) procedures. Nevertheless, these results must be taken in context. A propensity-matched analysis study [50] found that patients with UCS undergoing correction with springs were more likely to go to the ICU and have longer hospital stays when compared to those undergoing endoscopic craniectomy. Other large multi-center studies noted that endoscopic cranial remodeling procedures generally result in shorter procedures and ICU stays and lower utilization of blood [51].

A recent publication by Tevlin [52] presented endoscopic spring placement for UCS management as a way to reconcile these two procedures. Their cohort of 17 patients had an EBL of 45 ml and a mean procedure duration of 73 min. They had a 23.5% rate of major complications, mainly due to spring-related complications and suture refusion. This is still an early but promising technique, and the authors recognize that there are still technical refinements that need to be done. For instance, the authors point out that the bone indentations used for spring placement may help prevent the dislodgement they observed in 3 occasions. The endo-FODO technique currently performed at our institution demonstrates a favorable surgical profile, with an EBL of 9.4 ml/kg, a procedure duration of 104 min, and a low complication rate (24%). These findings align with prior studies and further support the safety profile of modern endoscopic approaches for the correction of UCS [40, 43, 50–52].

### Aesthetic outcomes

**Fig. 3:**
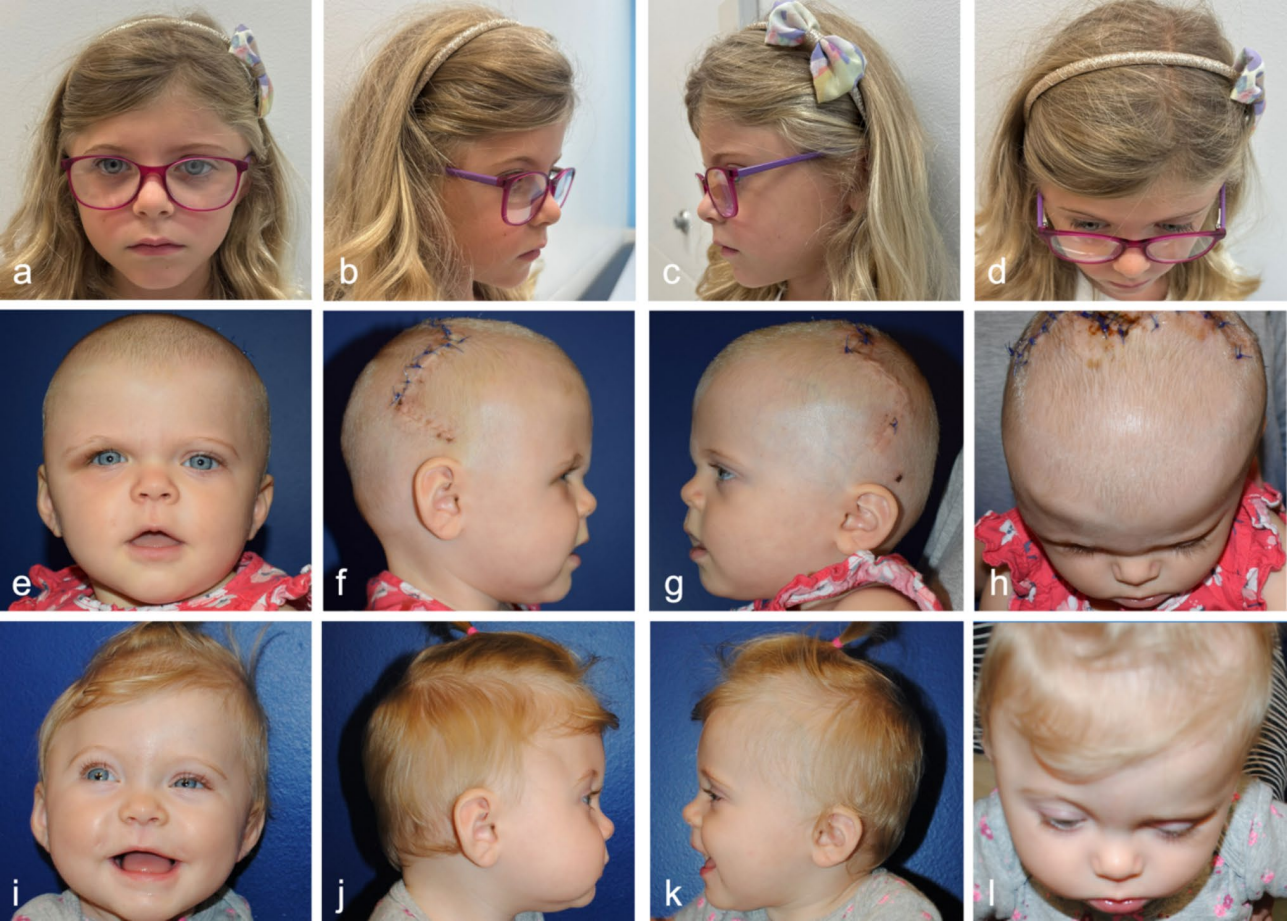
A female patient with right unicoronal craniosynostosis before undergoing FOAR at 10 months of age (*bottom row)*, at 1 month postoperatively (*middle row*), and at long-term follow-up 7 years postoperatively (*top row*)

A prior publication from our group [40] had shown the long-term aesthetic outcomes of FODO 5 years postoperatively. In that study, we had also reported a greater improvement in canthal tilt symmetry with FODO. In the present update, we found that canthal tilt symmetry improved by 1.5 degrees with FODO, while it worsened by 1.2 degrees with FOAR (*p* = 0.035). This suggests that FODO consistently provides a higher degree of improvement for canthal tilt after a median of over 7 years of follow-up (Fig. 3).

**Fig. 4:**
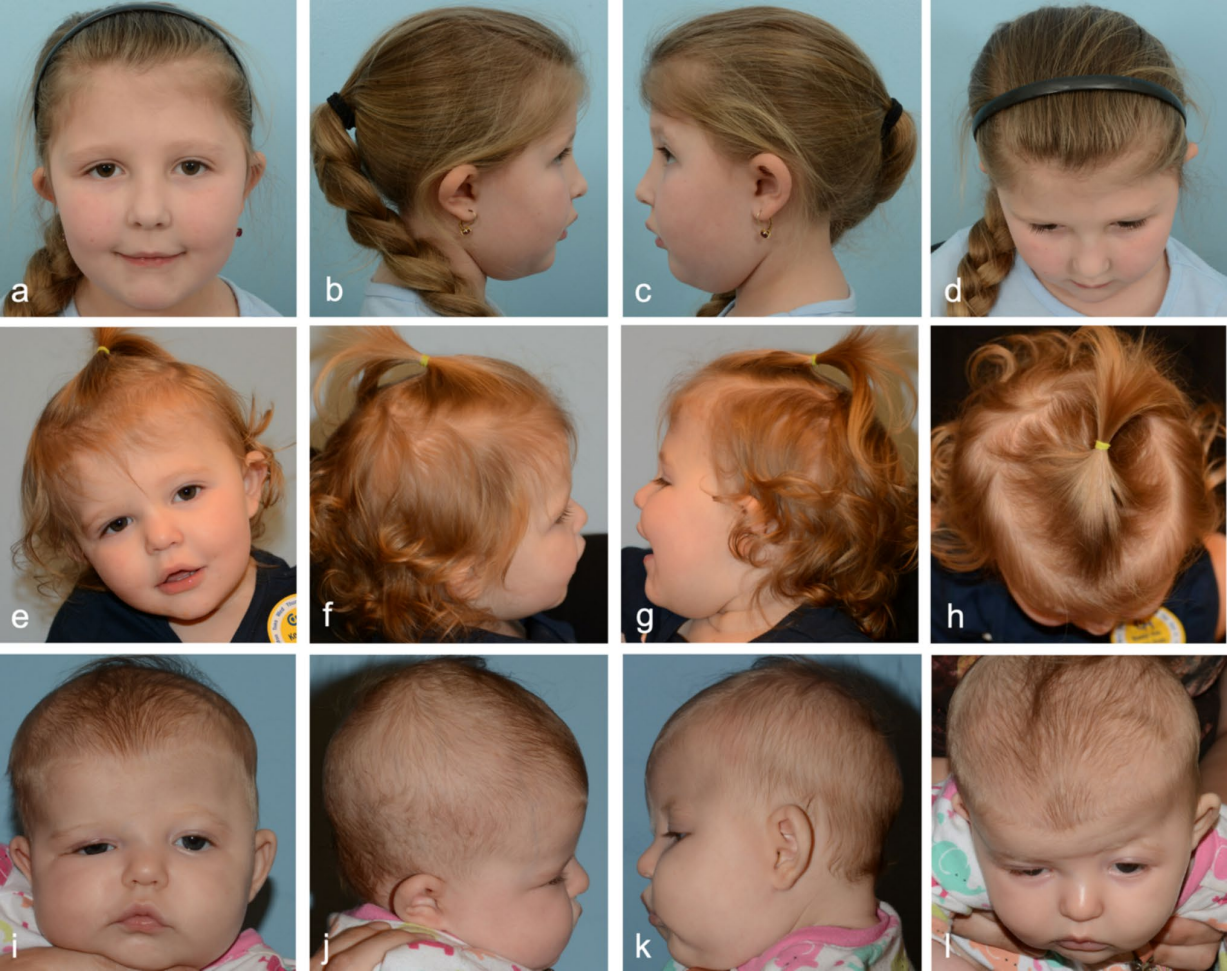
A female patient with left unicoronal craniosynostosis before undergoing open fronto-orbital distraction osteogenesis at 3 months of age (*bottom row*), at 9 months after distractor removal (*middle row)*, and at long-term follow-up 5 years after distractor removal (*top row*)

Our prior study found greater improvements in ODA angle in the FODO group [36, 37, 40], however, we did not find that same advantage for distraction in our current update (Fig. 4). Similarly, Mellgren and colleagues [36, 37] also found superior ODA correction when comparing FODO vs calvarial switch FOAR. They follow the same osteotomy pattern that we do for FODO. But their follow-up period was limited to 3 years. We believe the different findings may have been due to the progressive nature of UCS [13], which may have caused a diminishment of the ODA in this latest update. It must be noted that both FODO (88.4 degrees) and FOAR (87.5 degrees) had ODA angles close to 90 degrees, but the long-term changes in columellar base deviation may have rendered the differences between the groups insignificant. This also highlights the challenges of correcting the middle facial third asymmetry seen with UCS [13]. Further, this supports the idea that overcorrection may be required to counteract this progressive course of the UCS, as argued by other authors [53]. Additionally, Mellgren and colleagues found that the ipsilateral side continued to have smaller absolute orbital volume, even though FODO showed better orbital shape improvement.

In contrast, recent studies on spring-assisted craniectomy for UCS noted improvements in orbital volume and height, but not the same improvements in orbital width [49]. We also found that palpebral width symmetry had improvement after FODO (+ 0.32) compared to FOAR (−3.4). This underscores the challenge of addressing the horizontal orbital discrepancy seen in UCS regardless of technique. The group at Rady Children’s Hospital found no significant differences in the orbital morphology on the synostosed side when comparing FODO and FOAR [28, 54, 55]. They did find greater improvement on the contralateral side orbital volume through both procedures. It must be noted that their craniometric outcomes were limited to less than 26 months, which also limits the conclusions that can be drawn with respect to long-term outcome of their specific FODO technique.

We think that the specific osteotomy pattern and vector of distraction we utilize for FODO allows for correction of the orbital roof on the ipsilateral side, which directly improves the orbital height symmetry. Further, the unilateral distraction we perform may have the additional benefit of improving the orbital width of the contralateral side, which is likely what led to our improved outcomes in palpebral width, but we warn that these outcomes may not be obtained if other distraction techniques are used, as evidenced by Park and colleagues [38, 41], and other groups [28, 54, 55].

### Limitations

A major limitation of our study is the retrospective nature, which limits collection of standardized photographic data. We performed all measurements on 2D photographs since they were readily available on the patients’ electronic medical record. We acknowledge that 3D photography has become increasingly popular for evaluation of precise facial morphology since it introduces less bias and allows more precise facial symmetry evaluations [13, 33, 53]. However, we did not have 3D photographs available for the patients in our cohort and were therefore unable to perform this analysis. We attempted to minimize this source of error by including only photographs in which patients were facing straight into the camera and utilizing a previously employed photogrammetric measurement method. Additionally, younger patients had a shorter follow-up time and were therefore excluded from the long-term analysis. This limited the power of the analysis and the conclusions we could draw regarding the long-term outcomes of endo-FODO.

## Conclusion

In our comparison of FODO and FOAR for treatment of the UCS deformity, we report superior long-term periorbital symmetry with FODO along with a slightly higher infection rate. Other emerging treatment modalities reviewed but not included in this study, such as open and endoscopic spring-assisted cranioplasty, show similar promising results from dynamic procedures. Future studies will focus on utilizing 3D photogrammetric assessment and performing severity-matched analyses to better delineate some of the nuances found in our study.

## Data Availability

Data is available upon reasonable request.
